# Carroll A Campbell Jr Neuropathology Laboratory: Demystifying Alzheimer's Disease and Related Dementias Diagnoses in South Carolina

**DOI:** 10.1002/alz70855_107184

**Published:** 2025-12-24

**Authors:** Dorea P Jenkins, Laura Davis, Jennifer Belk, Michelle Ackerman, Shelby Carter, Eric D Hamlett, Steven L Carroll

**Affiliations:** ^1^ Medical University of South Carolina, Charleston, SC, USA

## Abstract

**Background:**

South Carolina has an extraordinarily high prevalence of Alzheimer's disease (AD) and AD‐related dementias (ADRDs), with approximately 122,699 individuals living with dementia. Mapping of ICD‐10 codes shows that these diseases are non‐uniformly distributed, with multiple “hot spots” in the state. Due to the complexity of these diseases, a post‐mortem examination of the brain is required for an accurate diagnosis. The Carroll A. Campbell, Jr. Neuropathology Laboratory (CCNL) at the Medical University of South Carolina (MUSC) has thus performed detailed histologic examination of 343 cases to define the causes of dementia in South Carolina, determine the prevailing ADRD type across SC and whether these deviated from national averages and establish whether specific kinds of neurodegenerative diseases were concentrated in the “hot spots”.

**Method:**

Donors were recruited via physicians, community outreach, and our website. Gross and microscopic examination of the brain was performed following consensus recommendations and using a panel of stains including hematoxylin and eosin (H&E), modified Bielschowsky stains, and immunostains for Abeta, hyperphosphorylated tau, alpha‐synuclein, p62, *p*‐TDP43, 3‐repeat and 4‐repeat tau. All cases were reviewed by a board‐certified neuropathologist. Diagnoses were mapped at a zip‐code level.

**Result:**

The 343 cases included 195 male and 148 female donors, with the average age of donors being 73.8 years. 148 cases (43.1%) had an AD diagnosis alone or in combination with another disease. 20.3% of the AD cases were early onset AD, as opposed to the usual 5‐10%. Frontotemporal lobar degeneration (FTLD)‐tau represented 63.6% of the FTLD cases (normally 45%) of cases, with FTLD‐TDP representing 26.4% of cases (normally 45%). Multiple system atrophy also occurred at a higher‐than‐expected rate. Pure AD was uncommon, with the majority of donors with AD having multiple neurodegenerative diseases. Mapping of these diagnoses indicated that certain types of neurodegenerative diseases clustered in specific regions of the state.

**Conclusion:**

The results of these autopsies suggest that South Carolina has a higher‐than‐expected representation of early onset AD and FTLD‐tau and that certain types of neurodegenerative disease cluster at specific locations. We are currently performing whole genome sequencing to determine whether specific disease‐causing genetic variants contribute to these diagnostic skews.

